# The Impact of Boron Nitride Additive on Thermal and Thermochromic Properties of Organic Thermochromic Phase Change Materials

**DOI:** 10.3390/ma17153632

**Published:** 2024-07-23

**Authors:** Natalia Paprota, Magdalena Szumera, Kinga Pielichowska

**Affiliations:** 1 Department of Biomaterials and Composites, Faculty of Materials Science and Ceramics, AGH University of Krakow, al. Mickiewicza 30, 30-059 Kraków, Poland; npaprota@agh.edu.pl; 2 Department of Ceramics and Refractory Materials, Faculty of Materials Science and Ceramics, AGH University of Krakow, al. Mickiewicza 30, 30-059 Kraków, Poland; mszumera@agh.edu.pl

**Keywords:** phase change materials, thermochromism, boron nitride, thermal conductivity, energy storage

## Abstract

Thermochromic phase change materials (TPCMs) are gaining increasing interest among scientists. These multifunctional materials can store thermal energy but also, at the same time, during the phase transition, they can change colour. Thermal conductivity is also extremely important for this type of material, which is why various additives are used for this purpose. This work aimed to study the properties of thermochromic phase change materials with an inorganic modifier. Stearic acid, behenyl alcohol, and bromocresol purple were used as thermochromic system components, while boron nitride particles were used as an additive. The key tests for such systems are thermogravimetric analysis (TGA) and differential scanning calorimetry (DSC), which allow determining the thermal stability of the materials (at around 170 °C) and phase transition parameters (thermal energy storage of 300 J/g in the range of 40–75 °C). The thermochromic properties were tested, and satisfactory results were obtained. In the end, laser flash analysis (LFA) tests indicated that boron nitride improves the thermal conductivity of the organic thermochromic phase change material by almost 30%. The results showed that the tested materials have great potential as thermochromic phase change materials for thermal energy storage.

## 1. Introduction

Green energy harvesting is rapidly advancing as a research field, leading to significant interest in efficient renewable energy storage solutions. Phase change materials (PCMs) are ideal for enhancing solar-powered devices for heat storage, potentially boosting the efficiency of thermal energy storage systems. Additionally, PCMs have various applications, including in buildings, electronics, and biomaterials. Thermochromic phase change materials (TPCMs) not only store thermal energy but also change colour during phase transition, making them a promising research area with considerable commercial potential. During phase transitions, phase change materials (PCMs) can absorb and release thermal energy. PCMs are classified into organic, inorganic, composite, and metallic. Organic phase change materials are, most of all, paraffin waxes, fatty acids, fatty alcohols, and poly(ethylene glycol) (PEG) [1,2]. In 2018, Li et al. [2] developed a thermochromic phase change material consisting of crystal violet lactone, stearic acid, and cetyl alcohol, with expanded perlite used as the encapsulating material. The synthesized samples exhibited favorable thermal and thermochromic properties. Among them, the (CVL)-10SA-30CA/EP MAR sample demonstrated the most promising results, with melting and solidifying enthalpies of 158.6 and 152.6 J/g, respectively, at melting and solidifying temperatures of 42.1 and 44.3 °C. Furthermore, the material changed color between blue and white at 42 °C. In 2023, Wu et al. [3] developed thermochromic microencapsulated PCMs for cold energy storage. Core materials were CVL–bisphenol A–n-tetradecane and n-dodecanol, with a melamine-formaldehyde resin shell. The TC-MPCMs, which contain 25 wt.% n-dodecanol and have a core/shell ratio of 1.85, exhibit an impressive encapsulation efficiency of 67.8%. Their thermochromic properties were satisfactory, with a colour difference of 23.39. The material operates effectively in a temperature range of 3–14 °C and has a melting enthalpy of 169.9 J/g. Additionally, it demonstrates excellent thermal stability at temperatures below 96 °C and outstanding thermal durability over 100 cycles. TPCM allows for the observation of color changes in materials during phase transitions, making the visualization of this process more intuitive and straightforward. This composition can effectively store latent heat and also directly indicate storage and energy consumption states through colour changes. Thermochromic phase change materials can directly reflect the energy saturation and depletion states in PCM applications through color changes. This could enhance its practical application value in latent heat energy storage, such as smart textiles, process temperature control, reversible thermochromic coatings, food and drug packaging, thermal sensors, and more [4].

However, in the case of organic PCMs, the thermal conductivity properties are not very satisfactory. Increasing the thermal conductivity can speed up the process of absorbing and releasing, thus improving the effectiveness of systems designed for thermal energy storage. There are essentially two approaches to improving thermal conductivity: incorporating materials known for their high thermal conductivity or using encapsulated phase change materials [5]. Microencapsulation is a good method as apart from the thermal conductivity enhancement, it can solve the problem of PCM leakage during the phase transition. Direct contact of PCMs with the environment is also restricted. The best materials for PCMs encapsulation with thermal conductivity enhancement properties are metals, ceramics, and even polymers with modifiers such as graphite oxide. On the other hand, encapsulation usually significantly decreases the latent heat of the phase transformation, therefore, the balanced ratios of the components must be selected. However, the addition of a high thermal conductivity modifier is the most commonly used method. Among the most effective additives, we can distinguish 3D foams (metallic or carbon), 2D layers (kaolin or expanded graphite), 1D additives (carbon nanotubes or fibers), and 0D particles (boron nitride, silicon nitride, and aluminum oxide) [5,6,7]. Metallic additives have high thermal conductivity, however, their high density and chemical reactivity often limit their utilization. Theoretically, carbon-based additives with the diversity of allotrope, low density, and thermal stability seem to be the best choice, but due to the black color, they may be limited in some applications, e.g., thermochromic materials. Similarly, as for encapsulation, the application of highly conductive additives usually reduces the PCM’s phase transition heat consequently it is necessary to limit their amount [8].

In recent years, multiple research focusing on improving the thermal conductivity enhancement was conducted. Wang et. al. [9] tested the thermal properties of the paraffin-based composites containing multi-walled carbon nanotubes. The thermal conductivity enhancement ratios were 35.0% in the solid state and 40.0% in the liquid state for the composite with a mass fraction of MWCNTs. The addition of the carbon modifier only slightly decreased the latent heat capacity by 1%. Bing et al. [10] proposed flexible PEG-based form-stable PCMs by taking cross-linked unsaturated polyester resin as the skeleton and expanded graphite as a thermal conductive additive. The composite with 7 wt.% of EG was characterized by high thermal conductivity (3.5 W/m⋅K which is an 1150% improvement over pure PEG). In 2014, Fang [11] et al. tested paraffin-based composite phase change materials filled with hexagonal boron nitride nanosheets (0, 1, 2, 5, and 10 wt.%). Research has shown that the thermal conductivity of PCMs, in both solid and liquid states, can increase to 60% as the content of h-BN nanosheets increases. Moreover, the melting and solidification points of these composite PCMs remained largely unchanged when h-BN nanosheets were added, while their latent heat of fusion decreased slightly with increased loading. Yung [12] did another research on boron nitride on thermal conductivity—both cubic and hexagonal boron nitride particles were used to prepare epoxy resin-based composites. Due to the use of silane surface treatment of fillers and multimodal mixing of particle sizes (using two different sizes of hexagonal particles and one size of cubic particles) before preparing the composite, the thermal conductivity of composites with an epoxy matrix filled with boron nitride increased by up to 217%.

A relatively novel approach to thermal conductivity enhancement is the infiltration of organic PCMs into the conductive aerogel. Wei et al. [13] tested PEG composites with microcrystalline cellulose (MCC)/graphene nanoplatelets (GNPs) aerogel. The thermal conductivity of the composite increased by more than 300% (from 0.31 to 1.03 W/m⋅K) compared to the MCC/PEG composite. In particular, the value of the phase transformation enthalpy remained largely unchanged, maintaining 99.84% of the value for pure PEG. Wang et al. [14] prepared nanoporous boron nitride aerogel film infiltrated with paraffin. The obtained composite was characterized by a thermal conductivity of 0.29 W/m⋅K which is 45% more than pure paraffin (0.2 W/m⋅K). Considering its great flexibility and shape stability, the BN aerogel-paraffin composite has great potential in advanced thermal management in portable electronics.

The materials studied fill the current literature gap. In the systems produced so far, attention was usually focused on only one aspect of the materials (PCM or thermochromism), which resulted in quite a random selection of individual components in terms of phase transformation temperatures. As a result, the colour change of the system occurred at a temperature not necessarily directly related to the PCMs phase change. By selecting acids and alcohols with similar phase change temperatures in this research, this problem was solved. Additionally, most of the systems tested so far were based on a different dye, i.e., crystal violet lactone and not bromocresol purple. Unlike previous research, the materials described in the article below do not appear as microcapsules but in bulk form. Although the influence of boron nitride on the thermal conductivity of organic materials is known, it was necessary to confirm a similar behavior in the case of the tested TPCMs and at the same time determine its influence on key properties for these types of materials, i.e., thermal and thermochromic properties. Due to these differences, it can be concluded that the tested materials are novel systems that can find applications in various fields.

This work aimed to study the properties of thermochromic phase change materials with an inorganic modifier. Stearic acid, behenyl alcohol, and bromocresol purple were used as components of the thermochromic system, while boron nitride particles were used as an additive to improve the thermal conductivity of TPCMs. A thermogravimetric analysis was performed to obtain data on the samples’ thermal stability, which is crucial for materials subjected to temperature changes during their applications. Differential scanning calorimetry is also an essential test for PCMs as it allows for determining the parameters of both melting and crystallization phase change processes. The thermochromic properties were tested and positive results were obtained. In the end, LFA tests enabled the analysis of the impact of the addition of boron nitride on the thermal conductivity of materials. The results showed that the tested materials have great potential as thermochromic phase change materials for thermal energy storage.

## 2. Materials and Methods

Merck KGaA provided bromocresol purple, stearic acid for synthesis, and behenyl alcohol for synthesis, while hexagonal boron nitride 500 nm and cubic boron nitride 165 nm were supplied by PlasmaChem GmbH (Berlin, Germany). All substances were utilized as received.

Based on the tests we conducted earlier, the TPCM with the BCP:SA:SA 1:10:40 ratio was selected as the one with the most optimal thermal and thermochromic properties. The amount of boron nitride introduced was adjusted based on data from the literature so that it had a positive effect on thermal conductivity and, at the same time did not deteriorate other thermal properties of the systems. The composition of the samples is presented in Table 1.

The mixtures of bromocresol purple (BCP), stearic acid (SA), and behenyl alcohol (BA) in a mass ratio of 1:10:40 were thoroughly ground in a mortar. The powder obtained this way was transferred into Petri dishes and then subjected to a vacuum oven at 80 °C. Boron nitride was added to the thermochromic phase change materials after melting. The samples were homogenized by sonication. The resulting solutions were then cooled in the air to form solid samples.

The Bruker Vertex 70V spectrophotometer (Billerica, MA, USA) was used to conduct infrared (IR) experiments in KBr pellets. The samples were scanned 64 times within the spectrum range of 4000 to 400 cm^−1^, with a resolution set at 2 cm^−1^.

Thermogravimetric analysis (TGA) was performed using the TGA 550 Discovery (TA Instruments, New Castle, DE, USA) thermobalance. Samples weighing approximately 5–6 mg were placed in uncovered platinum crucibles. The analysis was carried out within a temperature range of 40–600 °C, with a heating rate of 10 °C/min under a nitrogen atmosphere. From TG curves the temperatures corresponding to mass losses of 1%, 2%, 3%, 10%, and 50%, as well as from DTG curves temperatures of a maximum rate of mass loss T_DTGmax1_ and T_DTGmax2_ were determined.

Differential scanning calorimetry (DSC) measurements were carried out using a DSC1 (Mettler Toledo, Greifensee, Switzerland) calorimeter. Samples weighing around 4–6 mg were placed in sealed perforated aluminum crucibles. The analysis involved a heating → cooling → heating cycle within the temperature range of 10–90 °C. The heating rate was set at 10 °C/min and the measurement was carried out under a nitrogen atmosphere (30 mL/min).

DSC1 from Mettler Toledo was also used for modulated temperature DSC measurements (TOPEM mode). The measurement parameters included an amplitude of 1 K, a switching time of 15 s, and an underlying heating rate of 1 K/min within the temperature range of 10–75 °C.

The DSC isothermal step mode was used to generate curves of the stored heat as a function of temperature. The heat absorbed was methodically determined at each step and attributed to the temperature of the corresponding ramp phase. Subsequently, the heat curve Q(T) was constructed by adding the values from successive steps [15].

To analyze the color parameters of the samples, photographs were captured using a mobile phone (Samsung Galaxy A50, Suwon-si, Republic of Korea) at two temperatures: 25 °C (for solid samples post-cooling) and 75 °C (for liquid samples post-heating). These images were then transferred to a computer, and the L*, a*, and b* values were extracted using Adobe Photoshop CS5. Each recorded value was an average of four measurements taken at different locations. The colour was evaluated employing an independent CIE Lab model, where “L*” indicates luminance (with L* = 0 representing black and L* = 100 indicating white), “a*” signifies the range from green to magenta (negative values indicating green and positive values indicating magenta), and “b*” denotes the spectrum from blue to yellow (negative values indicating blue and positive values indicating yellow). ΔE* was calculated as:

(1) $$∆E=\sqrt{{{∆a}^{*}}^{2}+{{∆b}^{*}}^{2}+{{∆L}^{*}}^{2}}$$

where a*, b*, and L* are the color coordinates and Δa*, Δb*, and ΔL* are the respective changes in their values between the solid and liquid state for every sample. ΔE* is the color difference between samples at 25 °C and 75 °C, which is best suited to describing the color-changing process [16].

The laser flash analysis (LFA) was used to determine the samples’ thermal diffusivity of the tested using the Netzsch LFA 427 (Selb, Germany) analyzer. Each time during the measurement, the lower surface of the flat, parallelepiped sample was meticulously heated with a short laser pulse (20 J/pulse), with the having insulated side surfaces. The pulse caused the excitation of a heat source, which spread through the sample and caused a change in temperature inside the sample. As a result, the sample’s temperature change on its upper surface over time was recorded using an infrared detector. The rate of temperature equalization in the sample mass depends on its temperature diffusion coefficient, according to the following relationship:

(2) $$a=\frac{1.388\cdot h^{2}}{\tau_{0.5}}$$

where a is thermal diffusivity, mm^2^/s; h is sample thickness, mm; τ_0.5_ means time at 50% of the temperature increase, s [17,18].

The sample’s density and heat capacity at constant pressure must be known to determine the thermal conductivity coefficient based on the measured temperature diffusion coefficient. All samples were flat disks. The measurements were made at a temperature of 22 °C ± 2 °C. Detailed measurement parameters of the samples and their are presented in Table 2. 

The values of specific heat capacity and density for individual samples were determined based on the characteristics of the individual components of the mixtures and their mass ratios in the samples.

Taking into account the fact that thermal conductivity is a material-specific property used to characterize constant heat transport, its value was calculated using the following equation:

(3) $$\lambda \left(T\right)=\rho (T)\cdot c_{p}(T)\cdot a(T)$$

where: λ is thermal conductivity, W/m*K; ρ is sample density, g/cm^3^; a is thermal diffusivity, mm^2^/s; c_p_ is specific heat capacity, J/g*K [17,18].

## 3. Results and Discussion

**Figure 1:**
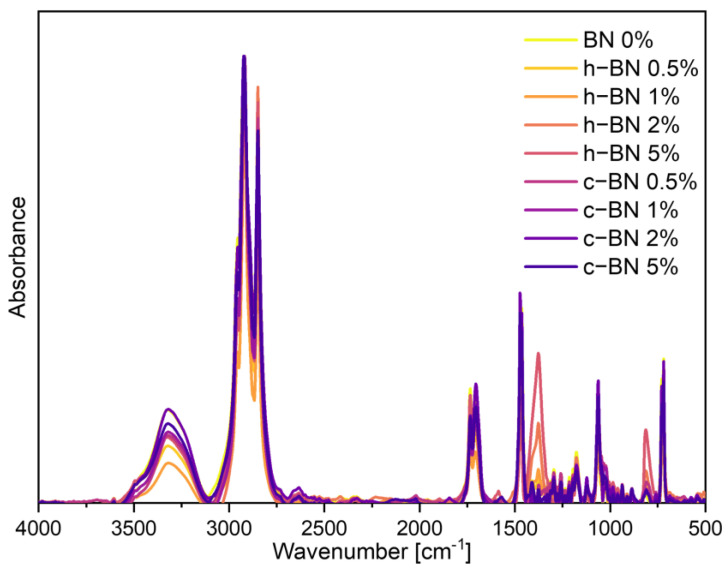
FTIR spectra of TPCMs mixtures.

FTIR spectra are presented in Figure 1 and data are collected in Table 3.

Examination of FTIR spectra verified the chemical composition of the TPCMs mixtures. Each spectrum exhibited bands typical of alcohols and acids, such as absorption of a hydroxyl group (OH) stretching vibrations (approx. 3322 cm^−1^), stretching vibrations of the carbonyl group (C=O) (1705 cm^−1^ and 1734 cm^−1^), C(O)-O acid stretching vibrations (1296 cm^−1^), OH out-of-plane bend (720 cm^−1^). Additionally, bands typical of organic compounds were observed including symmetrical and asymmetrical C-H stretching vibrations of CH_2_ methylene groups (2849 cm^−1^ and 2921 cm^−1^, respectively). Furthermore, absorption bands at 1174 cm^−1^ and 1063 cm^−1^ were linked to the structure of the benzene sulphonate group, indicating the opening of the ester ring group of bromocresol purple at room temperature. The boron nitride additive did not cause significant changes in the positions of the characteristic absorption bands. However, for samples with 1, 2, and 5% of the h-BN new absorption bands appeared at 1377 cm^−1^ and 811 cm^−1^ [19,20,21]. These results are consistent with research conducted by Ao et al. [22]. Their research was focused on stearic acid/boron nitride composites for TES and the same new bands appeared for samples with BN addition.

**Figure 2:**
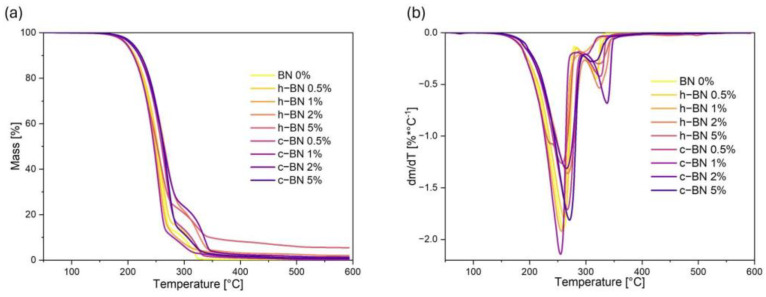
(**a**) TG and (**b**) DTG curves of TPCMs mixtures.

The thermogravimetric curves are presented in Figure 2; data are collected in Table 4.

The thermogravimetric analysis indicated that the materials obtained are thermally stable within the temperature range relevant to their potential applications, approximately 65 °C, which corresponds to the temperatures of phase transition. The temperature of 1% mass loss of materials is approximately 170 °C. Consequently, there exists a broad safe operational temperature range for these materials, enabling their utilization in real-time applications. The temperatures characteristic of thermochromic systems are slightly lower than those of the main base components, i.e., fatty acid and fatty alcohol. No significant dependencies were observed between the addition of boron nitride and the thermal stability of the samples. However, for samples with h-BN the mass residue increases, which means that the charred residue is the inorganic additive. Similar results of the influence of h-BN on the thermal stability of organic PCM were obtained by Su et al. [23]. In this research n–octadecane/stearic acid eutectic mixtures with hexagonal boron nitride were tested. TGA results are similar to ours and most importantly increasing charred residue is identified as the h-BN.

The TGA results also allow us to identify two stages of thermal degradation of TPCM mixtures [24]. In the first stage, approximately 80% of the material degrades at a lower temperature, while in the second stage, at a higher temperature, the remaining 20% decomposes. We can assume that in the first stage, the acid and part of the alcohol decompose, while at a higher temperature, the rest of the system degrades.

**Figure 3:**
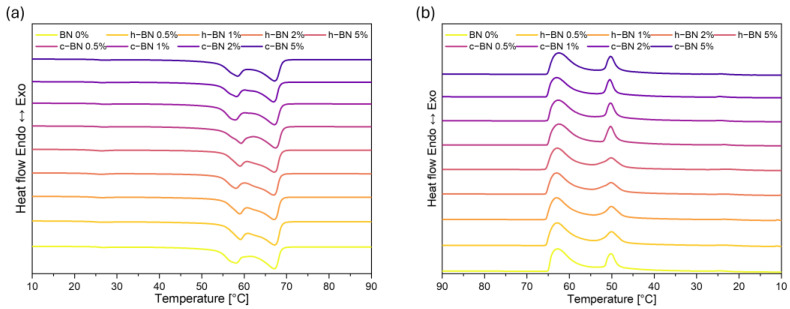
DSC heating (**a**) and cooling (**b**) curves of TPCMs mixtures.

Figure 3 presents DSC curves of TPCM mixtures, and data are collected in Table 5.

TPCM mixtures exhibit more than one endotherm in the DSC heating cycle. The first endotherm at a lower temperature is attributed to the eutectic phenomenon and appears at the eutectic temperature. This is typical of a eutectic system in which demixing and separation take place in the solid phase. The second endotherm at a slightly higher temperature is assigned to the liquidus temperature [25,26]. A similar situation occurs when materials are cooled and is attributed to crystallization processes. For melting processes, the eutectic temperature was recorded at 59 °C, while the liquidus temperature was 66 °C. In the case of crystallization, these temperatures are 50 and 63 °C, respectively.

It is observed that the addition of boron nitride does not significantly influence the temperatures of phase transitions. The shapes of the curves for all samples are almost identical. However, the amount of the heat of fusion and heat of crystallization decreased which is unfavorable but expected and consistent with other similar research (Ao, Su) [22,23]. In the case of PCMs, high enthalpy is crucial therefore, a compromise must be found between the amount of added modifier and the thermal properties of the samples.

Incorporating both fatty acid and fatty alcohol extends the duration of the phase transition (ΔT) to nearly 20 °C, whereas it remains below 10 °C for the base materials. This presents an advantage because the material can be used as an energy storage unit across a wider temperature range.

**Figure 4:**
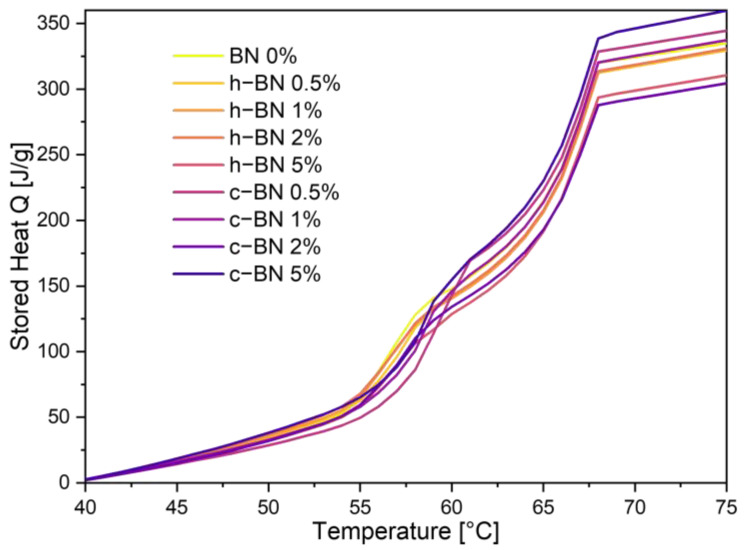
The stored heat of TPCMs mixtures during the heating process.

Figure 4 and Table 6 present the Q(T) curves of the mixtures and the results obtained using the step-mode DSC method.

Step-mode DSC was used to measure the amount of the stored heat during the melting process. The measurement took place from 40 to 75 degrees. Based on the results the TPCM mixtures can store significant amounts of thermal energy (over 300 J/g). It is visible that the amounts of stored heat depend on the modificator’s addition. Hexagonal boron nitride causes greater declines in the amount of stored heat with the higher amount of the modifier, while for cubic boron nitride samples surprisingly the stored heat even rises. The results also confirm that the phase change of these PCMs is not tied to a single temperature, but rather to a range of temperatures.

**Figure 5:**
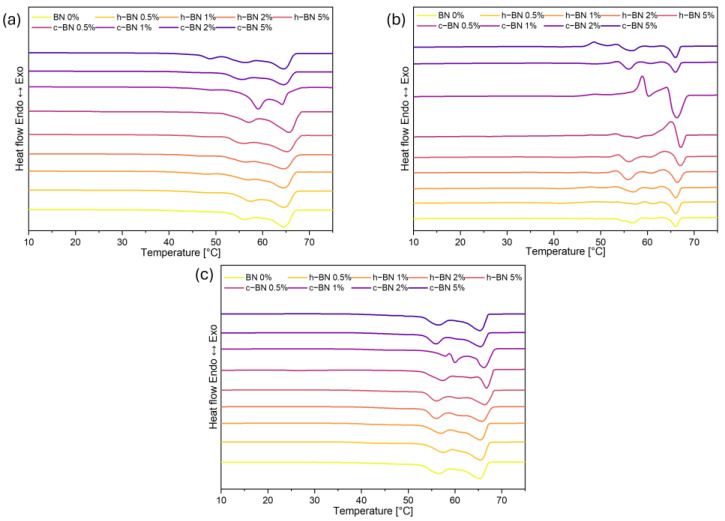
TOPEM DSC results: (**a**) reversing heat flow curves, (**b**) non-reversing heat flow curves, (**c**) total heat flow curves of TPCMs mixtures.

**Figure 6:**
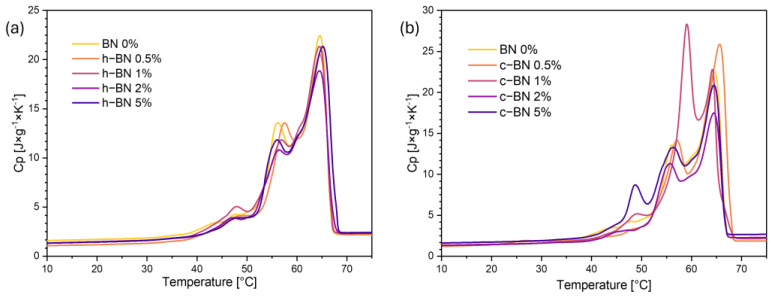
Specific heat capacity curves of TPCMs mixtures; (**a**) with hexagonal BN, (**b**) with cubic BN.

Figure 5 and Figure 6 present the TOPEM DSC results.

Reversing heat flow curves represent reversible melting during the heating process. In the nonreversing heat flow profile, it is possible to observe the exothermic peaks from recrystallization, which are followed by the peak attributed to the total melting. Samples with cubic boron nitride show more recrystallization, resulting in more defects in the crysta1line structure of materials. This may cause the heat of phase change to be lower. The total heat flow profiles are similar to those obtained by using conventional DSC. In most samples, we can see two separated peaks. Based on TOPEM results, we can conclude that cubic boron nitride has a greater impact on the thermal profiles of the materials than hexagonal boron nitride [27].

In Figure 6 are the specific heat capacity curves. These results confirm that the specific heat capacity changes during the phase transition. At the beginning of the measurement, the initial increase rate was small and along with the phase transition at 55 °C, the rising rate increased significantly. As in classic DSC curves, two separated peaks can be distinguished, which confirms that during the phase transition, the specific heat capacity increases sharply. Based on the curves of the change in specific heat capacity, the value of this parameter is slightly higher after the phase transition than it was before it [28]. The maximum specific heat capacity is about 20–25 J/g*K at around 65 °C. Based on picture (a) with h-BN we can say that this modifier did not cause significant changes in the curves’ shapes. On the other hand, in graph (b) where there are samples with cBN, we can conclude that the modifier significantly changed the shapes of the curves.

**Figure 7:**
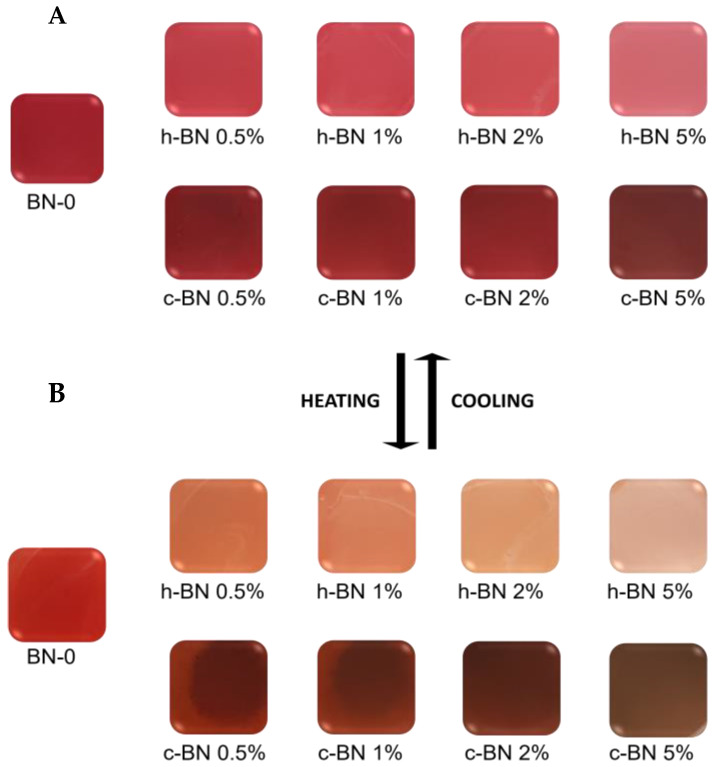
Digital photos of samples after the heating (liquid-(**B**)) and cooling (solid-(**A**)) process.

The thermochromic properties are presented in Figure 7.

These digital photographs of the samples confirm the thermochromic properties of the materials obtained. Liquid samples after heating are more orange or brown, whereas solid samples after the cooling process show a more pink color. The addition of boron nitride significantly influences the color of the samples. The hexagonal boron nitride powder is white, which causes lighter colors in the obtained samples. The cubic boron nitride powder is grey, so the samples are darker than the basic one without the modifier. The higher the amount of the modifier in the sample the more the difference in the color in comparison with the basic sample without the boron nitride.

**Figure 8:**
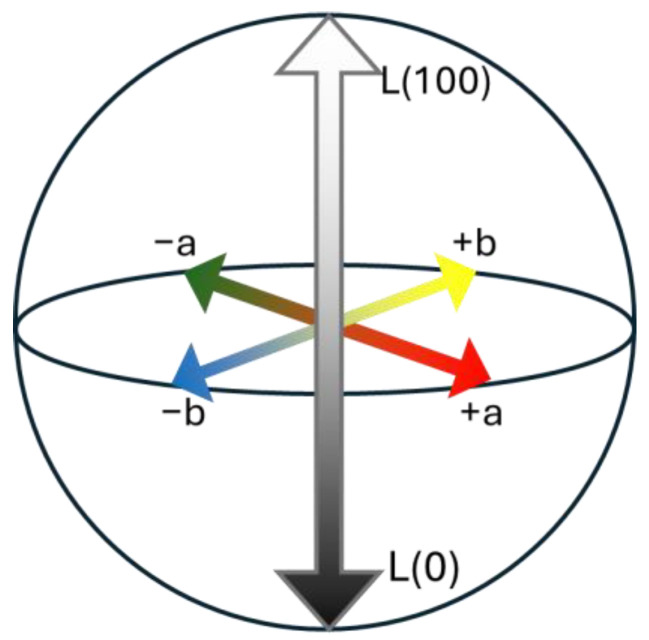
Three-dimensional CIE LAB color space where the L* axis represents the color’s lightness [29].

In order to determine the parameters of the colors according to the CIE LAB model (Figure 8) the computer software (Adobe Photoshop CS5) was used. Based on the digital photos the LAB values for every sample were determined in a solid and liquid state.

Table 7 contains numerical data of LAB values for TPCMs.

For the basic sample and those with hexagonal boron nitride, the lightness (L) of the samples increases after the melting process. The greater the amount of modifier in the sample, the greater the increase in brightness. On the other hand, for samples with cubic boron nitride, the lightness decreases. Both the parameters a and b are positive numbers as the colors of the samples are warm (more red and yellow than green and blue). In general, the parameter ΔE is the most suitable, as it describes the differences between the two colors. In this case, those are the colors in the solid and liquid states of the same sample. As the parameter ΔE is higher for samples with hexagonal boron nitride addition, we can state that these samples show greater differences in colors than those with cubic boron nitride addition.

The density, specific heat capacity, and thermal diffusivity were determined to obtain information on the thermal conductivity of the tested materials. All obtained results are presented in Table 8 and Table 9.

The LFA results enabled the determination of the thermal conductivity of the TPCM mixtures. It can be concluded that the thermal conductivity properties improve with increasing boron nitride content. The thermal conductivity value for a reference sample without BN additive is 0.252 W/m*K. The thermal conductivities of samples with hexagonal boron nitride rise from 0.284 W/m*K to 0.324 W/m*K when the loading changes from 0.5 mass% to 5 mass%, also for samples with cubic boron nitride, the thermal conductivity increases from 0.297 W/m*K to 0.326 W/m*K as the BN quantity rises from 1 mass% to 5 mass%. The value for the 0.5% c-BN sample is slightly overestimated compared to the other values, which may be because the measurement was carried out at a point where there was a higher concentration of boron nitride. Ultimately, we can conclude that both hexagonal and cubic boron nitride improve the thermal conductivity of the organic thermochromic phase change material by almost 30%. According to data from the literature, the thermal conductivity of the base ingredients is: stearic acid—0.3 W/m*K, behenyl alcohol—0.2 W/m*K, h-BN—600 W/m*K perpendicular to the c axis, c-BN—740 W/m*K [30,31,32]. Comparing obtained results with other literature data (Table 10) it can be concluded that obtained materials are characterized by satisfying thermal conductivity, higher than in many other research.

It is difficult to determine the application performance of the obtained TPCMs without specifying the actual application. However, we can assume that these materials are designed to operate in the temperature range of 45–70 °C. Based on this, we can confidently state that they are completely thermally stable in this temperature range. Furthermore, in this temperature range, phase transformations of melting and solidification also occur, and their colour changes, which is desirable and consistent with the initial assumptions.

## 4. Limitations of the Work and Future Scope

The main problem during the tests was the determination of color changes, due to the transparency of liquid TPCM. In further work, it would be beneficial to examine the temperature at which the colour of the material changes, for example, using a thermal imaging camera. This would allow us to confirm that the change occurs exactly within the temperature range of the phase transition. Another key element is the stabilization of the shape of TPCMs to limit the leakage of such material during phase transformations. For this purpose, porous materials could be used into which TPCMs could be infiltrated.

## 5. Conclusions

In this research, thermochromic systems were prepared using bromocresol purple, stearic acid, and behenyl alcohol. Various mass ratios of the boron nitride in hexagonal and cubic forms were used to improve thermal conductivity. Based on the results the following conclusions and comments can be drawn:

- The addition of boron nitride did not cause significant changes in the positions of the characteristic absorption bands. However, new absorption bands appeared at 1377 cm^−1^ and 811 cm^−1^ for samples with hexagonal boron nitride.
- The thermogravimetric analysis confirmed that the obtained materials are thermally stable in the temperature range corresponding to the potential applications, i.e., at the phase transition temperatures. TPCM mixtures show two-stage thermal degradation. The addition of boron nitride did not significantly influence the thermal stability of the samples.
- Samples are characterized by high enthalpies of phase transitions, which is crucial in thermal energy storage. The addition of boron nitride did not cause changes in the temperatures of phase transitions; however, in most samples, the heat of phase transitions dropped with the increased amount of the modificator. The step-mode DSC confirmed that during phase change material can store a high amount of thermal energy (over 300 J/g) but in most samples, the amount of stored heat dropped with the addition of the boron nitride. Therefore, it is crucial to select the right amount of modifier that will improve some other properties without causing too much heat loss.
- The determination of the colour parameters confirmed a clear change in the colour of the TPCM mixtures after the reversible processes of melting and solidification. The incorporation of boron nitride into samples caused the change in their colours (positive in the case of the hexagonal BN as the difference in these colours is greater and negative for the cubic one, because the colour difference is smaller). However, the thermochromic properties of the obtained materials are still satisfactory, since the colour change during the phase transition is visible.
- The LFA results confirmed that the addition of the boron nitride improved the thermal conductivity by almost 30% for samples with 5 mass% BN.
- Consequently, obtained thermochromic phase change materials exhibit great potential for thermal energy storage applications with additional indicator possibilities. They are characterized by high enthalpy of phase change enthalpy, good thermochromic reversible properties, and satisfactory thermal conductivity.

## Figures and Tables

**Table 1:** Composition of samples (mass ratio).

Sample Name	Bromocresol Purple [-]	Stearic Acid [-]	Behenyl Alcohol [-]	h-BN [Mass %]	c-BN [Mass %]
BN 0%	1	10	40	0	0
h−BN 0.5%	1	10	40	0.5	0
h−BN 1%	1	10	40	1	0
h−BN 2%	1	10	40	2	0
h−BN 5%	1	10	40	5	0
c−BN 0.5%	1	10	40	0	0.5
c−BN 1%	1	10	40	0	1
c−BN 2%	1	10	40	0	2
c−BN 5%	1	10	40	0	5

**Table 2:** LFA samples’ parameters.

Sample Name	Diameter [mm]	Thickness [mm]
BN 0%	12.8	2.09
h−BN 0.5%	12.52	1.94
h−BN 1%	12.51	1.81
h−BN 2%	12.85	1.85
h−BN 5%	12.48	1.67
c−BN 0.5%	12.46	1.57
c−BN 1%	12.50	2.09
c−BN 2%	12.30	1.83
c−BN 5%	12.38	1.70

**Table 3:** Interpretation of the FTIR spectra of TPCMs mixtures.

BN 0%	h–BN 0.5%	h–BN 1%	h–BN 2%	h–BN 5%	c–BN 0.5%	c–BN 1%	c–BN 2%	c–BN 5%	
3322	3322	3319	3323	3325	3320	3320	3320	3321	OH stretching (alcohol)
2924	2919	2918	2921	2919	2919	2921	2924	2921	CH_2_ asymmetric stretching
2849	2849	2849	2849	2849	2849	2849	2849	2849	CH_2_ symmetric stretching
1734	1734	1734	1734	1734	1734	1734	1734	1734	stretching vibrations of C=O (stearic acid)
1705	1705	1705	1705	1705	1705	1705	1705	1705	
1472	1473	1473	1473	1473	1473	1472	1472	1472	methylene C-H bend/C=C-C aromatic ring stretch
-	-	1377	1377	1377	-	-	-	-	h-BN
1296	1296	1296	1296	1296	1296	1296	1296	1296	C(O)-O stretching vibrations (stearic acid)
1177	1178	1178	1177	1176	1177	1178	1177	1177	structure of the benzene sulphonate group, suggesting that the ester ring group of BCP is opened
1063	1063	1063	1063	1062	1063	1063	1063	1063	
-	809	811	814	813	-	-	-	-	h-BN
720	720	720	720	719	720	720	720	720	OH out-of-plane bend (alcohol)

**Table 4:** TG results of TPCMs mixtures.

Sample Code	T_1%_	T_2%_	T_3%_	T_10%_	T_50%_	T_DTGmax1_	T_DTGmax2_	M_resid_
	[°C]	[°C]	[°C]	[°C]	[°C]	[°C]	[°C]	[%]
BN 0%	172	184	191	213	251	259	319	0.185
h–BN 0.5%	170	182	189	212	253	261	317	0.830
h–BN 1%	173	184	190	211	248	257	301	1.370
h–BN 2%	177	190	197	221	265	266	324	2.017
h–BN 5%	168	180	188	209	252	256	321	5.441
c–BN 0.5%	178	190	196	220	260	267	326	0.284
c–BN 1%	172	183	190	210	246	255	300	0.412
c–BN 2%	176	189	196	220	264	265	338	1.315
c–BN 5%	180	193	200	223	263	271	312	0.735
stearic acid	180	191	198	221	258	274	-	0.146
behenyl alcohol	185	196	203	228	270	287	-	0.055
bromocresol purple	244	247	249	256	557	257	-	48.409

**Table 5:** DSC results of TPCMs mixtures.

Sample Code	Tm_onset_ [°C]	T_m1_ [°C]	T_m2_ [°C]	Tm_endset_ [°C]	ΔT_m_ [°C]	Heat of Fusion [J/g]	Ts_onset_ [°C]	T_s1_ [°C]	T_s2_ [°C]	Ts_endset_ [°C]	ΔT_s_ [°C]	Heat of Crystallization [J/g]
BN 0%	54	58	66	69	15	194.22	65	63	51	48	17	186.06
h–BN 0.5%	54	59	67	69	14	185.90	66	63	51	47	19	178.27
h–BN 1%	55	59	66	69	14	186.97	66	64	50	47	19	179.89
h–BN 2%	54	58	66	69	15	175.89	66	64	50	47	19	169.90
h–BN 5%	55	59	66	69	14	179.06	66	63	50	47	19	172.72
c–BN 0.5%	54	59	67	69	16	184.30	65	63	51	45	20	177.98
c–BN 1%	53	57	66	69	16	189.83	65	63	51	45	20	182.73
c–BN 2%	53	58	66	68	16	163.33	66	63	51	45	21	160.30
c–BN 5%	53	58	67	69	16	192.34	65	63	51	45	20	186.71
stearic acid	68	–	71	75	7	201.77	68	67	–	62	5	207.51
behenyl alcohol	70	–	72	76	6	233.16	70	68	–	61	9	232.92

**Table 6:** The amount of stored heat in TPCMs mixtures.

Sample Code	Stored Heat [J/g]
BN 0%	334.86
h–BN 0.5%	329.55
h–BN 1%	330.93
h–BN 2%	330.70
h–BN 5%	310.55
c–BN 0.5%	344.52
c–BN 1%	337.31
c–BN 2%	304.41
c–BN 5%	359.87

**Table 7:** LAB values for samples after the heating (at 75 °C) and cooling (at 25 °C) process.

Sample	25 °C			75 °C			ΔE
	L*	a*	b*	L*	a*	b*	
BN 0%	36.00	50.75	27.75	40.25	55.00	42.00	15.47
h–BN 0.5%	46.50	52.75	26.50	55.50	36.25	38.00	22.03
h–BN 1%	49.00	52.75	24.50	61.00	33.75	34.25	24.50
h–BN 2%	51.00	50.75	24.75	68.25	22.75	33.75	34.10
h–BN 5%	56.75	42.75	16.00	72.75	16.00	19.25	31.34
c–BN 0.5%	31.00	42.50	23.75	26.00	35.50	28.25	9.71
c–BN 1%	29.25	39.75	23.75	25.75	29.50	25.50	10.97
c–BN 2%	30.00	40.00	23.25	21.75	22.50	19.50	19.71
c–BN 5%	28.25	28.75	16.75	30.00	15.25	19.25	13.84

**Table 8:** Parameters necessary to determine thermal conductivity.

Sample Name	Density [g/cm^3^]	Specific Heat Capacity [J/g*K]	Thermal Diffusivity [mm^2^/s]
BN 0%	0.872	1.694	0.171
h–BN 0.5%	0.879	1.690	0.191
h–BN 1%	0.885	1.687	0.196
h–BN 2%	0.898	1.677	0.203
h–BN 5%	0.935	1.652	0.210
c–BN 0.5%	0.885	1.686	0.216
c–BN 1%	0.898	1.686	0.196
c–BN 2%	0.924	1.675	0.177
c–BN 5%	0.997	1.648	0.198

**Table 9:** Thermal conductivity of TPCMs mixtures.

Sample Code	Thermal Conductivity [W/m*K]
BN 0%	0.252
h–BN 0.5%	0.284
h–BN 1%	0.313
h–BN 2%	0.306
h–BN 5%	0.324
c–BN 0.5%	0.324
c–BN 1%	0.297
c–BN 2%	0.274
c–BN 5%	0.326

**Table 10:** Comparison of thermal conductivity improvement of organic PCM by boron nitride.

PCM	BN Mass %	Thermal Conductivity Enhancement [%]	Ref.
stearic acid + behenyl alcohol	5	30	This study
paraffin	5	25	[11]
n-octadecane/stearic acid eutectic	5	6	[23]
PEG	5	40	[33]
paraffin	5	16	[34]

## Data Availability

The authors confirm that the data supporting the findings of this study are available within the article.
